# Investigation of Hexylamine Adsorption on Gold in Perchloric Acid

**DOI:** 10.3390/molecules28135070

**Published:** 2023-06-28

**Authors:** Gennady I. Ostapenko, Nina A. Kalashnikova

**Affiliations:** Medicinal Chemistry Center, Togliatti State University, 14 Belorusskaya St., 445020 Togliatti, Russia; nina.kalashnikova@tltsu.ru

**Keywords:** gold, surfactant, hexylamine, adsorption, cyclic voltammetry, chronoamperometry, EIS

## Abstract

The adsorption of hexylamine at the solution–gold interface in 1 M HClO_4_ in the presence of 0.1 M Fe^2+^ and 0.1 Fe^3+^ was studied by potentiodynamic, chronoamperometric and EIS methods. The main kinetic characteristics of the oxidation-reduction reaction iron ions (exchange current density, transfer coefficient, diffusion coefficients of iron ions) were determined. It was shown that the physical adsorption of hexylamine on gold can be described by the Dhar–Flory–Huggins isotherm. The values of the adsorption constant and the Gibbs free adsorption energy were obtained. A comparison of the free adsorption energy at these interfaces with the interaction energies of hexylamine and water molecules, and hexylamine molecules with each other was carried out. It was shown that hexylamine adsorption at all of these interfaces is due mainly to the hydrophobic effect of the interaction of hexylamine and water molecules.

## 1. Introduction

Adsorption is frequently one of the steps in many heterogeneous chemical processes. First of all, the adsorption mechanism determines the action of industrial adsorbents. In another example, many metals are catalysts, and the catalysis process includes an adsorption step on these metals. The mechanism of this stage largely determines the rate of catalytic reactions. In particular, it is convenient to study the mechanism and kinetics of redox catalytic reactions on metals by electrochemical methods. In this case, the elementary reaction act is accompanied by the flow of an electric current, which is easily quantitatively fixed.

Of particular interest is the adsorption of polar organic molecules (surfactants) on metal electrodes. The surfactant adsorption on electrodes regulates the processes of galvanization, corrosion, electrocatalysis, etc. to a large extent [1,2]. In particular, the surfactant adsorption determines the corrosion rate of metals when they are used as corrosion inhibitors. Therefore, the explanation of the adsorption mechanism is the most important task in the study of electrochemical kinetics.

In general, surfactant adsorption can occur by chemisorption or physical adsorption. Chemisorption takes place if the functional group of a surfactant organic molecule tends to chemical interaction with the electrode material. For example, chemisorption of mercaptoimidazole due to the covalent interaction of a sulfur atom with a metal [3,4,5] or chemisorption of benzotriazole due to the interaction of a nitrogen atom with a metal [6] takes place. The main features of chemisorption are: (a) a high electrode surface coverage by the adsorbate at relatively low adsorptive concentrations in the electrolyte bulk [7,8]; (b) a relatively high value of the free adsorption energy [9,10,11]; (c) slight dependence of adsorption on temperature, i.e., low adsorption activation energy [12,13,14].

Physical adsorption is characteristic of surfactants, the functional groups of which do not tend to interact chemically with metals. Physical adsorption is characterized by the following features: (a) a high surface coverage by the adsorbate is observed at relatively high adsorptive concentrations [12,15]; (b) a low value of free adsorption energy [16,17,18]; (c) a strong decrease in adsorption with increasing temperature [19], i.e., high adsorption activation energy [15,20,21].

Physical adsorption can occur due to the electrostatic interaction of surfactant molecules: (a) with the metal surface; (b) with polar water molecules. In the first case, the dipole of a surfactant molecule induces a charge redistribution on the metal surface, and adsorption is due to the interaction of the molecule dipole with its own imaginary image [16]. In the second case, adsorption is due to the hydrophobic effect, i.e., displacement of polar surfactant molecules onto the solution surface [22,23].

In numerous publications, the physical adsorption of surfactants on metals is explained by the interaction of the surfactant with the metal and the corresponding quantum chemical calculations are given. The hydrophobic effect as a cause of surfactant adsorption at the solution–electrode interface is considered very rarely (see, for example, Ref. [24]), although the works [25,26] indicate a significant influence of the hydrophobic effect on surfactant adsorption. The need arises to quantify the contribution of the hydrophobic effect to the overall pattern of adsorption on metals.

Identification of the causes and adsorption mechanism is of scientific and practical interest. In the vast majority of works on the study of metal corrosion, the nature of adsorption is judged by the value of the free adsorption energy $\Delta G_{ad}^{0}$ (the so-called “20/40 criterion” [27]). It is assumed that during chemisorption, $\Delta G_{ad}^{0}$ < −40 kJ mol^−1^, and at physical adsorption, $\Delta G_{ad}^{0}$ > −20 kJ mol^−1^ (for example, see Ref. [27]). However, in Ref. [28] it is shown that this criterion is not reliable for several reasons. More reliably, physical adsorption and chemisorption can be identified theoretically by modeling the surfactant molecule–surface distance or by analyzing the electronic structure of the surfactant molecule–surface bond. In practice, this can be done spectroscopically, because the interaction of a surfactant molecule with the surface should distort the electronic configuration of the molecule [28].

In the study of adsorption on metals, chemisorption can be identified by the above signs, except for the 20/40 criterion. However, the physical adsorption mechanism (metal interaction or hydrophobic effect) is more difficult to identify. This is explained by the fact that the signs of physical adsorption listed above are characteristic of both mechanisms of such adsorption.

The study of adsorption on soluble metal electrodes (for example, on steel) seems to be quite difficult, because many phenomena of an electrochemical nature affect this process. The corrosion inhibition of steel by surfactant adsorption is a typical example. The main thing is that corrosion occurs at a stationary, but not equilibrium, potential. Hence, the following problems arise: (a) effect of the crystal lattice destruction during the dissolution of iron interferes with the study of adsorption undoubtedly; (b) along with the metal dissolution reaction, depolarization reactions simultaneously occur (reduction of H^+^ or OH^−^, usually), which complicates the analysis of kinetic patterns; (c) abundant gas evolution during depolarization reactions also interferes with the study of adsorption in a “pure” form. Therefore, the study of surfactant adsorption on an electrode at an equilibrium potential and the absence of a metal dissolution process is of interest. For these reasons, it is advisable to study the adsorption process on an inert electrode.

The main idea of this work is the following. If the parameters of surfactant adsorption at different interfaces (in particular, different metals) differ, then the cause of adsorption will be direct surfactant interaction with the adsorbent surface. If these parameters are close, then the lateral interactions of the solution components are the cause of adsorption. Here, hexylamine adsorption was studied on an inert gold electrode. Then, the obtained adsorption parameters were compared with the adsorption parameters of hexylamine on platinum [29] and the air–solution interface [29].

Here, adsorption of hexylamine on gold in an acid medium was studied in the presence of Fe^2+^ and Fe^3+^ ions. The presence of the redox couple Fe^3+^/Fe^2+^ makes it possible to reliably fix the equilibrium electrode potential.

Hexylamine was chosen as a surfactant because it has rather high surface activity [30] and low chemical activity of the amino group under the conditions studied here. Note that hexylamine is protonated in an acidic medium and is present in solution as a hexylammonium ion:


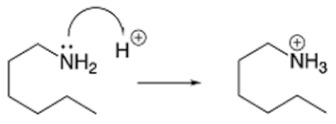


It is known that the effective electrode surface decreases during surfactant adsorption, and the exchange current density decreases in proportion to the electrode surface coverage by the adsorbate [31]. Therefore, the oxidation-reduction reaction of iron ions:

Fe^2+^ − e ↔ Fe^3+^ (1)

inhibited here by surfactant adsorption.

Perchloric acid was used as a solvent. First, it forms a weak complex with Fe^3+^ ions and practically does not form a complex with Fe^2+^ ions [32,33]. Therefore, the effective concentration of uncomplexed iron ions practically coincides with the total concentration. Second, perchlorate ions are practically not adsorbed on inert metals [34,35]. Therefore, the study of surfactant adsorption on gold is simplified. Under such conditions, i.e., in the practical absence of a ligand that forms a complex with iron ions, reaction (1) will proceed through aqua complexes [36]:

Fe(H_2_O)_6_^2+^ − e ↔ Fe(H_2_O)_6_^3+^. (2)

When Reaction (2) proceeds, the stage of diffusion of ions to the electrode takes place. Therefore, the kinetics of this reaction on gold is preliminarily studied by chronoamperometric and potentiodynamic methods to separate the charge transfer overvoltage from the diffusion overvoltage to determine the exchange current density. The charge transfer resistance values (related to the exchange current) are obtained by the electrochemical impedance spectroscopy (EIS) method. Next, the effect of surfactant adsorption on the value of the exchange current and charge transfer resistance is studied.

## 2. Results and Discussion

### 2.1. Cyclic Voltammetry

**Figure 1:**
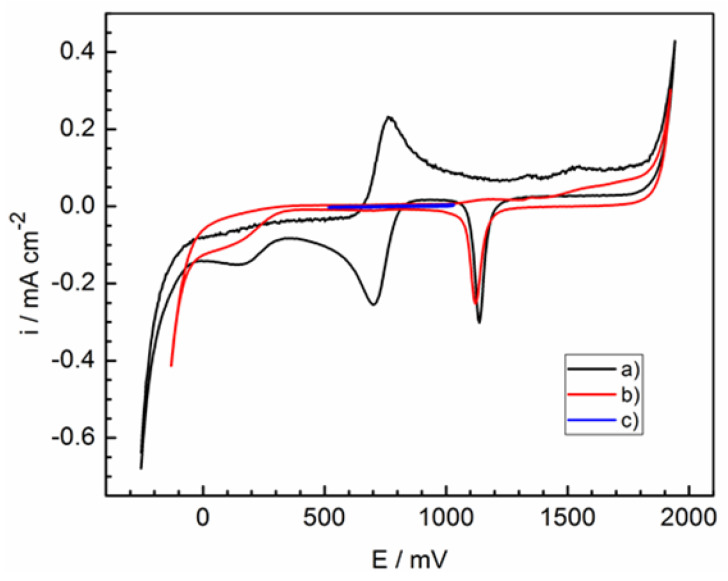
Cyclic voltammograms: (**a**) in background electrolyte (1 M HClO_4_), (**b**) 1 M HClO_4_ + 0.1 M hexylamine, (**c**) during electrode activation in solution (b). Potential sweep rate 20 mV s^–1^.

The experimental value of gold electrode equilibrium potential for the blank solution is 729 ± 2 mV relative to the standard hydrogen electrode. This value is in good agreement with the calculated potential value of 733 mV, taking into account the low complex formation in solution. During the polarization of inert electrodes in aqueous solutions, processes associated with the realization of hydrogen and oxygen electrodes take place. In Figure 1, the potentiodynamic curve (a) is shown for a background solution (1 M HClO_4_) without surfactant and iron ions. This curve is typical for inert electrodes in aqueous solutions. Here, at potentials less than −0.2 V, the reduction of protons occurs with the formation of gaseous hydrogen. At potentials of more than 1.8 V, the oxidation of hydroxide ions occurs with the formation of gaseous oxygen. Intermediate peaks characterize the formation–decomposition of surface oxides [37,38] and traces of formed oxygen and hydrogen.

New peaks do not appear in the presence of hexylamine (curve (b)). Therefore, hexylamine is electrochemically stable under given conditions. Significant electrochemical reactions are not observed in the potential range 0.5–1.0 V during electrode activation (curve (c)).

**Figure 2:**
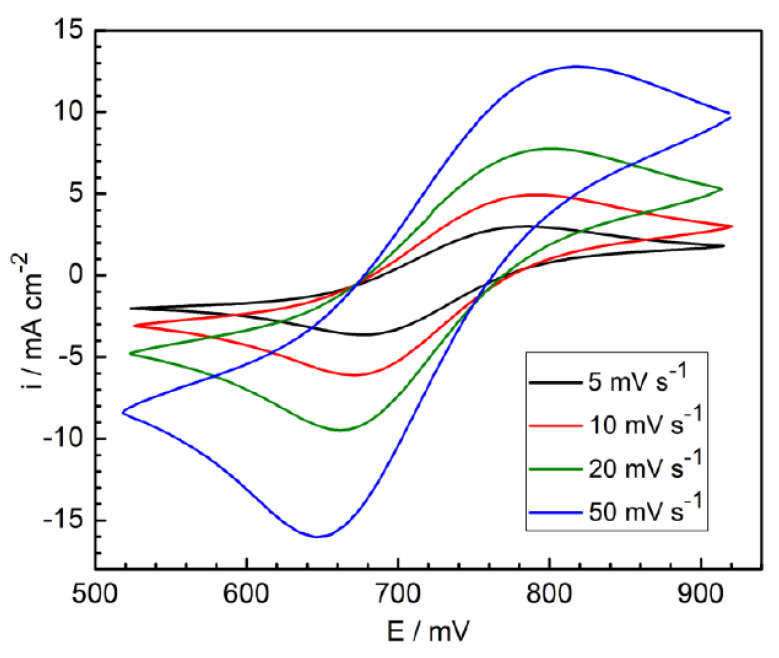
Cyclic voltammograms at various potential scan rates for the blank solution without hexylamine.

**Figure 3:**
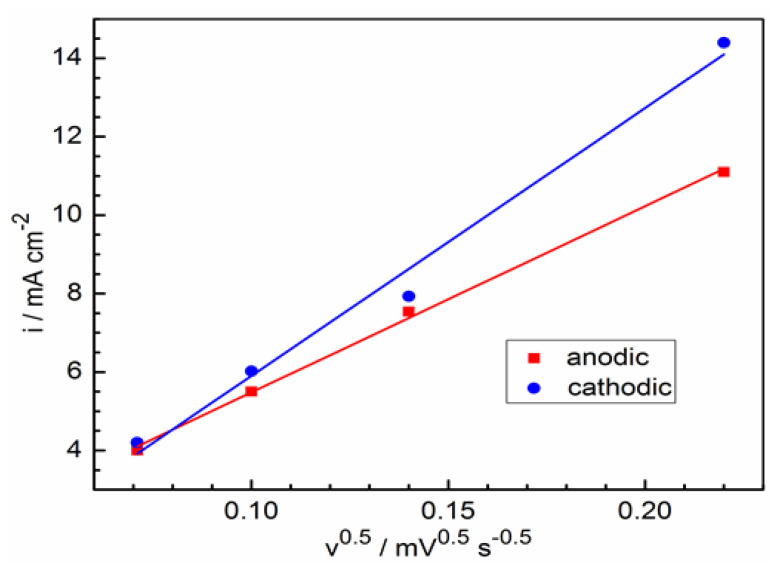
Dependence of voltammogram maximum currents on potential scan rates from Figure 2.

In Figure 2, cyclic voltammograms are shown for blank solution (in the presence of iron ions without surfactant) at various potential sweep rates. The observed currents of the iron ion oxidation-reduction are almost two orders of magnitude higher than the background currents for oxygen and hydrogen (see Figure 1), and therefore they can be neglected. These curves are typical of a reversible electrochemical reaction under diffusion control. In this case, the peak currents are described by the Randles–Shevchik equation [39,40,41]:

(3) $$i(p)=0.446\sqrt{\left[\frac{nF}{RT}\right]}nFc\sqrt{D}\sqrt{v}$$

where *n* is the ion valence change in the reaction, *F* is the Faraday constant, *R* is the gas constant, *T* is the temperature, *c* is the molar concentration of iron ions, *D* is the ion diffusion coefficient, *v* is the potential scan rate. In Figure 3, the dependencies of the anode and cathode maxima currents are given as coordinates determined using Equation (3). These dependencies are satisfactorily extrapolated to zero coordinates, and give $D_{Fe^{2+}}$ = 3.9 × 10^−6^ cm^2^ s^−1^ for anodic maxima and $D_{Fe^{3+}}$ = 5.6 × 10^−6^ cm^2^ s^−1^ for cathodic maxima. These values agree satisfactorily with the results of other works (see Table 1).

### 2.2. Chronoamperometry

**Figure 4:**
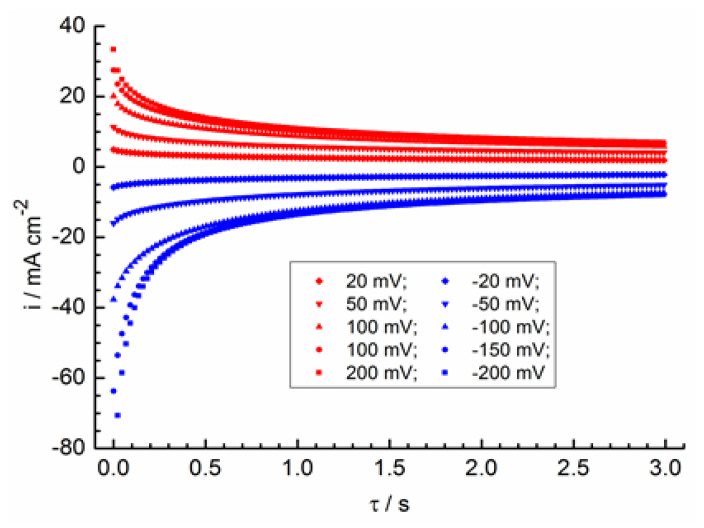
Chronoamperograms at various overvoltages for a blank solution without hexylamine.

In Figure 4, chronoamperograms are shown at various potentials for a blank solution without surfactant. These chronoamperograms are typical for diffusion kinetics and are described by the Cottrell equation at elevated potentials [44,45]:

(4) $$i(\tau )=nF\sqrt{\frac{D}{\pi }}c\left(\exp\frac{nF}{RT}\eta -1\right)\frac{1}{\sqrt{\tau }},$$

where *i*(τ) is the current density, η is the overvoltage, and τ is time.

**Figure 5:**
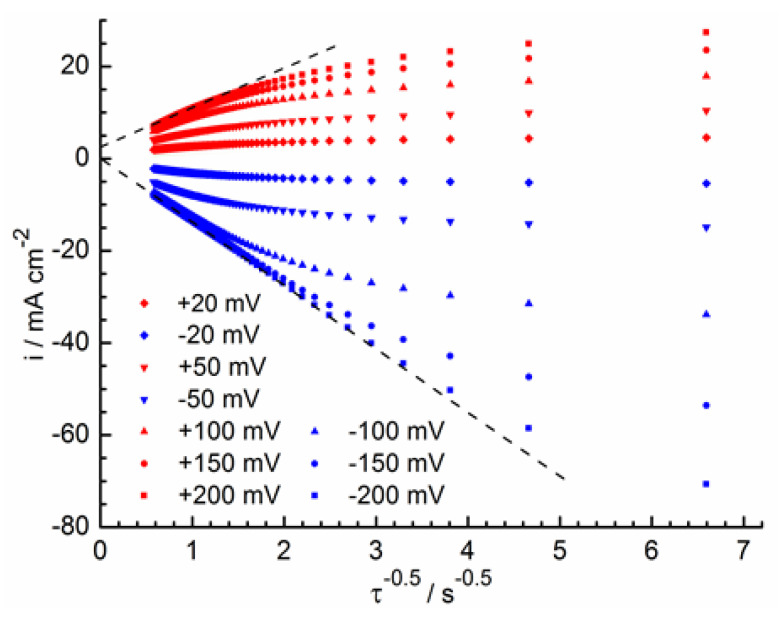
Chronoamperograms from Figure 4 in the coordinates from Equation (4).

In Figure 5, the chronoamperograms are shown in coordinates obtained using Equation (4). The straight sections of chronoamperograms should be extrapolated to zero coordinates with semi-infinite diffusion. Some non-fulfillment of this condition during anodic polarization can be explained by a significant electrostatic interaction of the formed Fe^3+^ ions with each other and with the components of the solution due to the higher charge of these ions compared to Fe^2+^. The diffusion coefficient of Fe^3+^ is $D_{Fe^{3+}}$ = 6.2 × 10^−6^ cm^2^ s^−1^, which is in accordance with the slope of the cathode chronoamperograms at elevated cathode overvoltages. A less reliable estimate of the Fe^2+^ diffusion coefficient gives a value of about $D_{Fe^{2+}}$ = 2.9 × 10^−6^ cm^2^ s^−1^, according to the slope of the anode chronoamperograms. The obtained *D* values do not contradict the results of other works on the Reaction (2) kinetics in a perchlorate medium (see Table 1).

Extrapolation of chronoamperograms to zero time gives currents due only to the kinetics of charge transfer. These currents are described by the Butler–Volmer equation:

$$i(0)=i_{0}\left\{\exp\left[\frac{\alpha nF}{RT}\eta \right]-\exp\left[-\frac{(1-\alpha )nF}{RT}\eta \right]\right\},$$

where *i*_0_ is the exchange current density, and α is the transfer coefficient. It is convenient to use this equation in the form proposed by Allen and Hickling [46]:

(5) $$\ln\frac{i(0)}{1-\exp\left[-\frac{nF}{RT}\eta \right]}=\ln i_{0}+\frac{\alpha nF}{RT}\eta ,$$

which is linear for all overvoltages.

**Figure 6:**
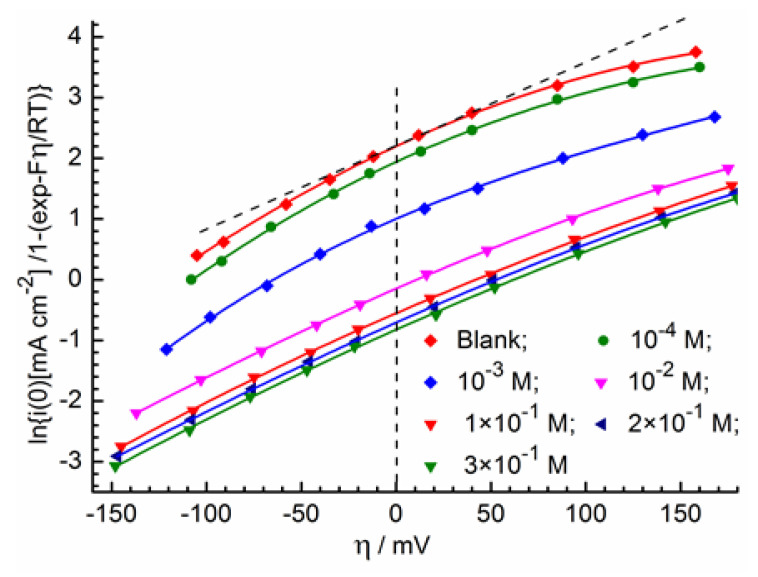
Dependence of electron transfer currents on overvoltage at various hexylamine concentrations in Equation (5) coordinates.

In Figure 6 (blank curve), the dependence of the extrapolated charge transfer currents on overvoltage is given in the coordinates obtained using Equation (5) for the solution without surfactant. This dependence was plotted taking into account the voltage drop in the electrolyte of 0.48 Ω cm^2^ (according to EIS measurements). Some nonlinearity in the experimental dependence is apparently due to the problems of the chronoamperogram extrapolation to zero time at high overvoltages. The exchange current density for a blank solution is 9.0 mA cm^−2^. The value of α estimated tangentially at zero overvoltage is 0.57 (at low overvoltages, the problems with extrapolation are minimized).

The standard rate constant of charge transfer reaction is estimated as follows [47]:

k0=i0nFcOx1−αcRedα,

where *c_Ox_* is the concentration of the ion oxidized form (here Fe^3+^), *c*_Re*d*_ is the concentration of the reduced ion form (here Fe^2+^). Here, *k*^0^ = 9.3 × 10^−4^ cm s^−1^ for blank solution. This value somewhat exceeds the values obtained in other works (see Table 2). The reasons for this excess are beyond the scope of this work and require a separate study.

In Figure 6, the dependences of transfer currents on overvoltage are also shown for solutions with different hexylamine concentrations. In Table 3, the exchange current densities and the corresponding charge transfer resistances are calculated from the following equation

$$R_{ct}=\frac{RT}{Fi_{0}}$$

for the indicated solutions. Obviously, the exchange current decreases and the charge transfer resistance increases with increasing surfactant concentration due to adsorption.

### 2.3. EIS Method

**Figure 7:**
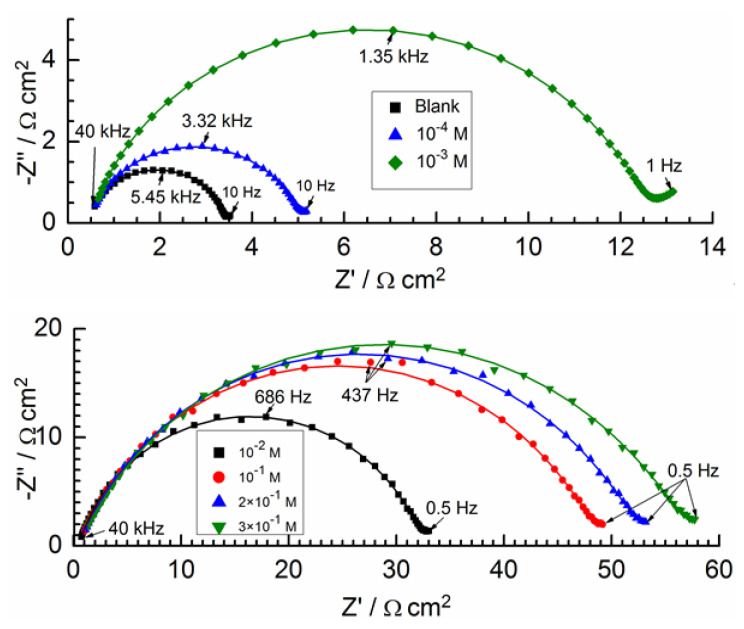
Nyquist plots for various hexamine concentrations.

**Figure 8:**
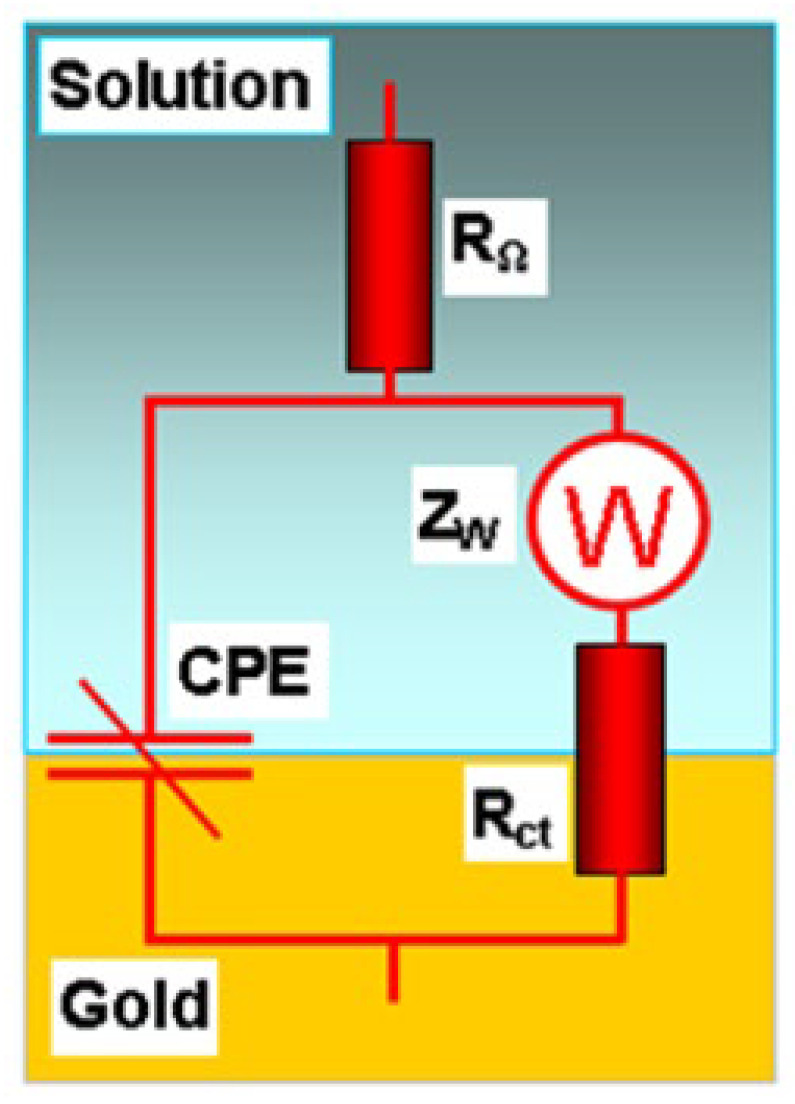
Equivalent electrical circuit for the solution–gold interface: R_Ω_ is the electrolyte resistance, CPE is the frequency-distributed double-layer capacitance, Rct is the charge transfer resistance, and Z_W_ is the Warburg diffusion element.

In Figure 7, Nyquist plots are shown for solutions with different hexylamine concentrations. These plots are modeled satisfactorily by the equivalent circuit shown in Figure 8. Here, CPE has the meaning of a frequency-distributed double-layer capacitance [50,51]. The CPE impedance is:

$$Z(CPE)=A^{-1}{\left(i\omega \right)}^{-n},$$

where *A* is a proportionality coefficient, ω is the angular frequency (in rad s^−1^) and i^2^ = −1 is the imaginary number; *n* is the exponent related to the phase shift and can be used as a measure of the surface inhomogeneity [52,53]. For the whole numbers *n* = 1, 0, −1, CPE is reduced to the classical elements capacitor (*C*), resistance (*R*) and inductance (*L*), respectively. Other values of *n* approximately describe other types of frequency distribution behavior of *C*, *R* or *L* with distributed parameters. The double layer capacitance is related to the *Z*(*CPE*) parameters by the equation [54]:

$$C_{dl}={\left(A\times R_{ct}{}^{1-n}\right)}^{1/n}.$$

In Table 4, the parameters of the equivalent circuit are given according to the data presented in Figure 7. From this table, it can be seen that the charge transfer resistance *R_ct_* increases and the *C_dl_* decreases with increasing surfactant concentration. This is explained by a decrease in the effective electrode surface with increasing surfactant adsorption.

### 2.4. Adsorption Isotherm

To quantify adsorption and construct an adsorption isotherm, it is necessary to determine the electrode surface coverage θ with adsorbate. Usually, when studying corrosion, θ is equated with the inhibition efficiency *IE*:

$$\theta =IE=1-\frac{i_{inh}}{i}=1-\frac{R}{R_{inh}},$$

where *i_inh_* and *R_inh_* are the current and polarization resistance in the surfactant presence, *i* and *R* are for a blank solution without surfactant. However, studies of electrode surfaces coating by X-ray photoelectron spectroscopy (XPS) [55] have shown that this method of calculating θ is incorrect, and therefore is unsuitable for quantitative calculations. The use of *IE* to calculate θ leads to a significant error in the Gibbs adsorption energy estimation [56].

Let us justify another method for surface coverage estimating from electrochemical measurements. In general cases [57]:

(6) $$\theta =\frac{\Gamma }{\Gamma_{max}},$$

where Γ is the actual excess adsorbate concentration on the surface, and Γ*_max_* is the adsorbate concentration on the surface at the maximum surface coverage. Upon adsorption, the effective solution–metal interface area decreases. This leads to a decrease in the exchange current density or to an increase in the charge transfer resistance *R_ct_* during electrochemical measurements. Here, the charge transfer resistance is more convenient for determining θ, because *R_ct_* is proportional to adsorbate concentration. Then, the “excessive” transfer resistance will be equal to *R_ct_* − *R_ct_*(0), where *R_ct_* is the actual value of the charge transfer resistance, *R_ct_*(0) is the charge transfer resistance for a blank solution (here, the appearance of *R_ct_*(0) is due to the fact that *R_ct_* ≠ 0 for *c_S_* = 0). In this case, Equation (6) can be rewritten as [29]:

(7) $$\theta =\frac{R_{ct}-R_{ct}(0)}{R_{ct}(\max)},$$

where *R_ct_*(max) is the charge transfer resistance at the maximum coverage of the electrode surface by the adsorbate.

Experimental determination of *R_ct_*(max) is impossible due to the low solubility of hexylamine in the background solution. However, in experiments on the study of surface tension at the interface between air and the test solution, the value Γ*_max_* = (7.0 ± 1.4) × 10^−6^ mol m^−2^ was determined [29]. Let us assume that the surface coverage θ is the same for the air–solution and electrode–solution interfaces at the same hexylamine concentration. For example, at *c_S_* = 0.1 M for the air–solution interface Γ = (4.36 ± 0.04) × 10^−6^ mol m^−2^ [29]. For the gold–solution interface from the chronoamperometric data, *R_ct_* = 45 Ω cm^2^ and *R_ct_*(0) = 2.9 Ω cm^2^ (Table 3) for the same surfactant concentration. Under these conditions, we equate the right parts of Equations (6) and (7) and obtain *R_ct_*(max) = 66 Ω cm^2^ for chronoamperometric measurements. For EIS measurements, *R_ct_* = (47.9 ± 2.5) Ω cm^2^ at *c_S_* = 0.1 M. Hence, *R_ct_*(max) is (71 ± 4) Ω cm^2^, which is close to the analogous value for chronoamperometric measurements.

In Table 5, surface coverage is given for chronoamperometric and EIS measurements at various hexylamine concentrations calculated according to Equation (7).

Surfactant adsorption can be represented as a process of displacement of water molecules by surfactant molecules at the interface [58,59]:

Surf_aq_ + *x*H_2_O_ad_ ↔ Surf_ad_ + *x*H_2_O_aq_, (8)

where the index “aq” characterizes the state of the molecule in the volume of the solution, the index “ad”—at the interface, *x* is the number of water molecules replaced by one surfactant molecule at the interface.

The problem of choosing an adsorption equation from many known ones arises when constructing an adsorption isotherm. For example, a detailed analysis of various equations is given in the Refs. [60,61,62]. However, it was shown by Bastidas that in the case of hexylamine adsorption, the Flory–Huggins equation is optimal [30]:

$$\frac{\theta }{x{\left(1-\theta \right)}^{x}}=K_{ad}c_{S}$$

or the refined Dhar–Flory–Huggins equation [63]:

(9) $$\frac{\theta }{e^{x-1}{\left(1-\theta \right)}^{x}}=K_{ad}c_{S},$$

where *K_ad_* is the adsorption constant. (Note that Equation (9) at *x* = 1 gives the Langmuir adsorption isotherm [64,65]). This equation can be rewritten in a form convenient for graphical analysis:

(10) $$\log\frac{\theta }{e^{x-1}{\left(1-\theta \right)}^{x}}=\log K_{ad}+\log c_{S}.$$

For hexylamine in aqueous solutions *x* ≈ 3 [29,30]. Then, Equation (10) for the system under study can be rewritten as:

(11) $$\log\frac{\theta }{e^{2}{\left(1-\theta \right)}^{3}}=\log K_{ad}+\log c_{S}.$$

**Figure 9:**
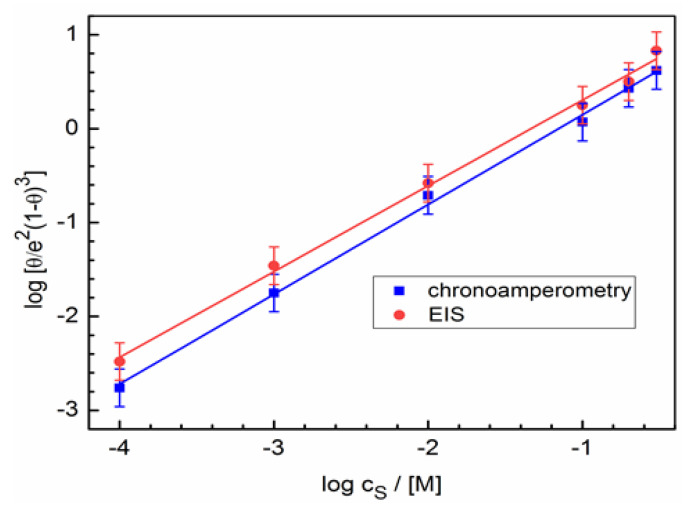
Dhar–Flory–Huggins isotherms for hexylamine adsorption on gold from chronoamperometric and EIS measurements at the coordinates obtained using Equation (11).

Adsorption isotherms for chronoamperometric and EIS measurements are plotted in Figure 9 at the coordinates obtained using Equation (11). Extrapolation of the straight lines to log *c_S_* = 0 gives the values of the adsorption constant *K_ad_*. The Gibbs free adsorption energy is related to the adsorption constant [66,67]:

$$\Delta G_{ad}=-RT\ln\left(55.5K_{ad}\right),$$

where 55.5 is the molar concentration of water in the solution.

Table 6 shows the main parameters of the hexylamine adsorption at the solution–gold interface, as well as for comparison, the corresponding parameters for the solution–platinum and solution–air interfaces [29].

Table 6 shows that the slopes of the straight line for both measurement methods on gold correspond satisfactorily to each other. The slope values are in good agreement with the theoretical unit slope as well. This confirms the assumption that the adsorption of hexylamine on the studied metals is well described by the Dhar–Flory–Huggins isotherm. The relatively low adsorption free energy (less than −20 kJ mol^−1^) confirms the physical nature of hexylamine adsorption, although this is discussed in [28].

In the general case, the free energy of the hexylamine adsorption can characterize the processes: (1) direct physical interaction of the solution components with the metal surface and (2) lateral interaction of the solution components.

The first group includes physical interaction with the gold surface:

(a) water molecules ($\Delta G_{H_{2}O-\mathrm{Au}}$= (−20…−30) kJ mol^−1^ [68]);
(b) hexylamine molecules ($\Delta G_{\mathrm{HA}-\mathrm{Au}}$).

The second group includes the interaction:

(c) water molecules with each other via hydrogen bonds ($\Delta G_{H_{2}O-H_{2}O}$ ≈ −21 kJ mol^−1^ [69]);
(d) hexylamine molecules among themselves ($\Delta G_{\mathrm{HA}-\mathrm{HA}}$ ≈ −25 kJ mol^−1^ [70,71]);
(e) hexylamine molecules and water ($\Delta G_{\mathrm{HA}-H_{2}O}$ = (−8.4…−12.5) kJ mol^−1^ [70,71]);
(f) water molecules and ${\mathrm{ClO}}_{4}^{-}$ ions ($\Delta G_{H_{2}O-{\mathrm{ClO}}_{4}^{-}}$ ≈ (−290…−340) kJ mol^−1^ [72]).

Without taking into account lateral interactions, direct (a) and (b) interactions can characterize the direction of Reaction (8). The $\Delta G_{\mathrm{HA}-\mathrm{Au}}$ value is unknown, but it can be assumed that this value should be slightly higher than $\Delta G_{H_{2}O-\mathrm{Au}}$, because the equilibrium of Reaction (8) is shifted to the right. Some ${\mathrm{ClO}}_{4}^{-}$ ions affect hydrogen bonds in water [72] and, accordingly, the $\Delta G_{H_{2}O-H_{2}O}$ value must also be taken into account here.

Incidentally, the absence of ${\mathrm{ClO}}_{4}^{-}$ ion adsorption on metals can be explained by the very high energy of the (e) interaction between water molecules and these ions.

Obviously, the value of the resulting energy of hexylamine adsorption on gold will be determined by the total energy effect of these interactions, mainly lateral interactions. Quantitative accounting of all interaction types is difficult. However, at a qualitative level, some assumptions can be made. Obviously, for the solution–air interface, adsorption is predominantly due to the hydrophobic effect. Therefore, the similarity of the $\Delta G_{ad}^{0}$ values for the solution–gold, solution–platinum, and solution–air interfaces suggests that the hexylamine adsorption on these metals is also due mainly to (e) interaction, i.e., the hydrophobic effect. The energy $\Delta G_{\mathrm{HA}-H_{2}O}$ of hydrophobic interaction of hexylamine molecules with water is about of 10 kJ mol^−1^. The experimental $\Delta G_{ad}^{0}$ values are about of 17 kJ mol^−1^ for both metals (see Table 6). Apparently, about of 7 kJ mol^−1^ (17 kJ mol^−1^−10 kJ mol^−1^) remaining from the experimental total energy characterizes some participation of (b) interaction, because some structuring of surfactant molecules near the platinum surface can be observed even in oil media [73]. At elevated θ, (d) interaction can also appear.

Therefore, we can conclude that the hexylamine adsorption at various interfaces has a common nature and is mainly due to the hydrophobic effect of the displacement of surfactant molecules onto the solution surface, regardless of the interface nature (solution–metal or solution–air).

### 2.5. Temperature Dependence of Adsorption

In Table 7, the Reaction (2) parameters are summarized at different temperatures and hexylamine concentrations according to EIS measurements.

**Figure 10:**
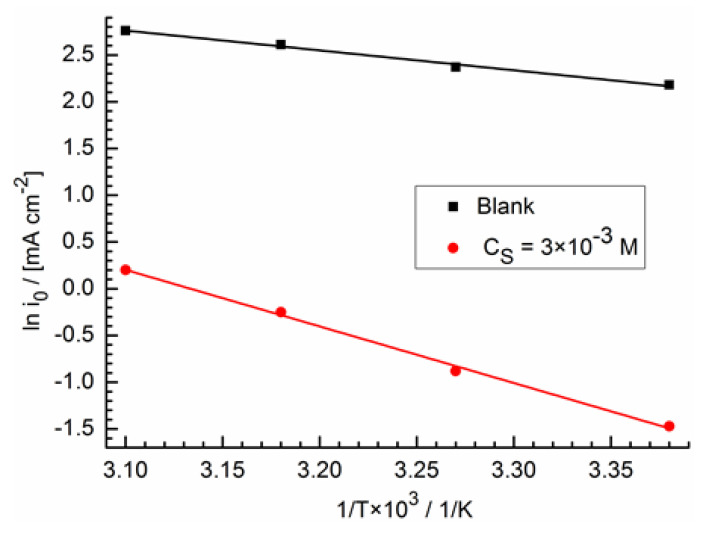
Dependence of the density exchange current on the hexylamine concentration at different temperatures at the coordinates obtained using Equation (12).

The dependence of the exchange current density on temperature is given by the Arrhenius equation:

$$i_{0}=B\exp\left(-\frac{E_{a}}{RT}\right)$$

or:

(12) $$\ln i_{0}=\ln B-\frac{E_{a}}{RT},$$

where *B* is a constant, *E_a_* is the adsorption activation energy. Figure 10 was produced according to the data obtained using Equation (12) and presented in Table 7. From the straight line slope, it follows that *E_a_* = (17.2 ± 3.0) kJ mol^−1^ for a solution without surfactants and *E_a_*_,HA_ = (49.7 ± 3.2) kJ mol^−1^ at a surfactant concentration of 0.3 M.

Such a high activation energy value in the presence of surfactant shows a strong adsorption weakening with increasing temperature, which is typical for physical adsorption [12,13,74]. Therefore, in our case, the physical nature of the hexylamine adsorption on the gold is confirmed. Physical adsorption is also characteristic of hexylamine adsorption on steel in an HCl solution [75].

## 3. Experimental Section

### 3.1. Preparation of Solutions

To prepare the basic solution without surfactants (blank solution) of the composition 1 M HClO_4_ + 0.1 M Fe^3+^ + 0.1 M Fe^2+^, metallic iron powder was dissolved in HClO_4_ to form a solution of Fe(ClO_4_)_2_. Then, the solution was filtered and H_2_O_2_ was added to oxidize a part of Fe^2+^ to Fe^3+^ [76]. The amounts of reagents and solutions were selected in such a way as to obtain the same concentrations of Fe^2+^ and Fe^3+^ 0.1 M in 1 M HClO_4_ solution. The purity of all reagents was at least 99.5%. The iron content in the samples was determined by the photometric method with sulfosalicylic acid [77]. The measurements were carried out at a wavelength λ = 510 nm.

Working solutions were obtained by adding the required amounts of hexylamine to the blank solution.

### 3.2. Equipment

Electrochemical measurements were carried out using a potentiostat-impedance meter IPC-ProM device (manufactured by the Institute of Physical Chemistry and Electrochemistry of the Russian Academy of Sciences). The experiments were carried out in a three-electrode glass cell with a glassy carbon counter electrode and the saturated silver chloride reference electrode. The Luggin capillary was used to eliminate the resistance between the working electrode and the reference electrode. All experiments, except those specifically indicated, were carried out at a temperature of 23 °C. The temperature was maintained with an error of 0.5 °C.

The working electrode was a gold foil with an area of 1 cm^2^. The electrode was degreased with a mixture of CaO + MgO and ethyl alcohol. Before measurements, the electrode was exposed in 1 M HClO_4_ for at least an hour.

### 3.3. Electrochemical Measurements

Preliminary measurements showed that the electrode kinetic parameters slowly drift with time. Therefore, for reproducibility of the results, the electrode was activated, and measurements were taken 10 min after activation. This time is sufficient for carrying out the pre-starting procedure before the experiment. At the same time, the electrochemical parameters practically do not differ from the initial values after this time.

Activation is a common practice in the study of the electrochemical kinetics on inert electrodes [37,39,40,41,48,49]. Activation is carried out by potentiodynamic cycling of the electrode in the range from the hydrogen formation potential at cathodic polarization to the oxygen formation potential at anodic polarization. Here, activation was carried out by means of three potential scans at a rate of 20 mV s^−1^ within ϕ_0_ ± 200 mV, where ϕ_0_ is the equilibrium electrode potential.

EIS measurements were carried out in the frequency range of 40 kHz to 0.5 Hz at a voltage amplitude of ±10 mV Ac.

### 3.4. Estimation of Measurement and Calculated Values Errors

Here the results of one typical series of chronopotentiometric and potentiodynamic measurements were analyzed. Therefore, the magnitude of the random error was not taken into account. With EIS, measurements were taken at least three times under the same conditions with an estimate of the random error. The error in the calculated values was determined as the sum of the errors made in the measurements used for the calculations.

## 4. Conclusions

(1) A methodology for the investigation of the surfactant adsorption at equilibrium potential on inert electrodes by introducing a redox pair into the solution was proposed. The presence of this pair makes it possible to fix the electrode equilibrium potential and evaluate adsorption by changing the exchange current of the inhibited pair during adsorption.
(2) A method for calculating the surface coverage in electrochemical measurements at low surfactant solubility using the results of measurements of adsorption at the air–solution interface was proposed.
(3) The redox reaction of iron ions on gold was studied by potentiodynamic, chronoamperometric and EIS methods. It was found that the reaction occurs under diffusion control. The main kinetic characteristics of the reaction—the standard electron transfer rate constant and the diffusion coefficients of iron ions in solution—were obtained. The values of these characteristics did not contradict the results of other works.
(4) Hexylamine adsorption on gold was studied. It was shown that this process can be well described by the Dhar–Flory–Huggins isotherm equation for the number of displaced water molecules by the adsorbate molecule *x* ≈ 3. The slope of the straight lines is close to unity in the coordinates of this isotherm. This confirms the correctness of the choice of this equation for the experimental data analysis. The main characteristics of the adsorption process: adsorption constant, adsorption free energy and adsorption activation energy are obtained.
(5) The main adsorption characteristics on gold are compared with similar adsorption characteristics on platinum and the solution–air interfaces. The values of these characteristics for hexylamine adsorption at all interfaces are close, and are typical for physical adsorption. A comparison of the experimental Gibbs adsorption energy for these interfaces with the known energies of direct and lateral interactions showed that the cause of surfactant adsorption at these interfaces is predominantly the hydrophobic effect of the interaction of surfactant molecules with water molecules.
(6) The temperature dependence of the adsorption of hexylamine on gold has been studied. The high value of the activation energy confirms the physical nature of adsorption.

## Figures and Tables

**Table 1:** Diffusion coefficient values of iron ions in HClO_4_ solutions.

Ion	Ion Concentration, M	Acid Concentration, M	*D* × 10^6^, cm^2^ s^−1^	Reference	Research Method
Fe^2+^		1.0	6.5	[42]	Rotating disc electrode (RDE)
	10^−2^	1.4	4.2	[42]	RDE
			5.7	[42]	RDE
	10^−1^	1.0	2.9	This work	Potential step relaxation (PSR)
	10^−1^	1.0	3.9	This work	Cyclic voltammetry (CV)
Fe^3+^		1.0	5.5	[42]	RDE
	10^−2^	1.4	6.2	[42]	RDE
			6.5	[43]	RDE
	10^−1^	1.0	6.2	This work	PSR
	10^−1^	1.0	5.6	This work	CV

**Table 2:** Standard rate constant values of Reaction (2) on gold in HClO_4_ solutions.

Acid Concentration, M	α	k^0^, cm s^–1^	Reference	Research Method
0.5	0.41	3.3 × 10^−5^	[48]	PSR
0.5	0.59	3.2 × 10^−5^	[48]	PSR
0.5	0.5	5.0 × 10^−5^	[37]	RDE
0.5		4.0 × 10^−5^	[36]	PSR
0.5	0.59	8.0 × 10^−5^	[49]	RDE
1.0	0.57	9.3 × 10^−4^	This work	PSR

**Table 3:** Parameters of Reaction (2) according to chronoamprometic measurements.

*C_S_*, M	*i*_0_, mA cm^−2^	*R_ct_*, Ω cm^2^
blank	9.0	2.9
1 × 10^−4^	6.8	3.8
1 × 10^−3^	2.6	10
1 × 10^−2^	0.83	29
1 × 10^−1^	0.57	45
2 × 10^−1^	0.54	48
3 × 10^−1^	0.48	53

**Table 4:** Parameters of the Reaction (2) equivalent circuit according to EIS measurements.

*c_S_*, M	*R_ct_*, Ω cm^2^	*A* × 10^5^, s^n^ Ω^−1^ cm^−2^	*n*	*C_dl_*, μF cm^−2^	*W*, Ω s^−0.5^
blank	2.9 ± 0.3	2.20	0.93	10.6	1.99
1 × 10^−4^	4.5 ± 0.4	3.67	0.88	11.2	2.87
1 × 10^−3^	12.0 ± 1.0	4.05	0.85	10.5	3.26
1 × 10^−2^	31.7 ± 2.0	4.24	0.82	9.9	3.90
1 × 10^−1^	47.9 ± 2.5	4.66	0.77	7.5	4.50
2 × 10^−1^	51.9 ± 3.0	4.80	0.76	7.2	4.82
3 × 10^−1^	56.4 ± 3.0	4.84	0.74	6.1	5.4

**Table 5:** Surface coverage θ according to various research methods at different hexylamine concentrations.

Hexylamine Concentrations, M	Research Method	
	Chronoamperometry	EIS
1 × 10^−4^	0.013	0.023 ± 0.011
1 × 10^−3^	0.10	0.128 ± 0.033
1 × 10^−2^	0.37	0.41 ± 0.06
1 × 10^−1^	0.59	0.63 ± 0.07
2 × 10^−1^	0.63	0.69 ± 0.09
3 × 10^−1^	0.71	0.75 ± 0.09

**Table 6:** Main parameters of hexylamine adsorption at various interfaces.

Interface	Slopes of the Straight Line	Adsorption Constant *K_ad_*, L mol^−1^	Adsorption Free Energy ∆G^0^_*ad*_, kJ mol^−1^
Solution–gold:			
Chronoamperometry	0.96 ± 0.05	14.1 ± 4.0	−16.4 ± 1.1
EIS	0.92 ± 0.07	15.8 ± 5.0	−16.7 ± 1.2
Solution–platinum, EIS	0.94 ± 0.09	10.0 ± 4.0	−14.8 ± 5.0
Solution–air	0.98 ± 0.18	15.8 ± 0.3	−16.7± 0.3

**Table 7:** Exchange current density of Reaction (2) at various temperatures and hexylamine concentrations.

Interface	Slopes of the Straight Line	Adsorption Constant *K_ad_*, L mol^−1^	Adsorption Free Energy ∆G^0^_*ad*_, kJ mol^−1^
Solution–gold:			
Chronoamperometry	0.96 ± 0.05	14.1 ± 4.0	−16.4 ± 1.1
EIS	0.92 ± 0.07	15.8 ± 5.0	−16.7 ± 1.2
Solution–platinum, EIS	0.94 ± 0.09	10.0 ± 4.0	−14.8 ± 5.0
Solution–air	0.98 ± 0.18	15.8 ± 0.3	−16.7± 0.3

## Data Availability

The data presented in this study are available upon request from the corresponding author.
